# Extensive Soft Tissue Swelling in the Larynx and Hypopharynx of a Young Female Patient After Haloperidol Use: A Case Report on Angioneurotic Edema

**DOI:** 10.7759/cureus.41684

**Published:** 2023-07-11

**Authors:** Bamidele O Johnson, Nkolika Odenigbo, Denis Tcedilin, Patrice Fouron, Mahidul Chowduary

**Affiliations:** 1 Psychiatry and Behavioral Sciences, Interfaith Medical Center, New York City, USA; 2 Psychiatry, Interfaith Medical Center, New York City, USA; 3 Internal Medicine, Interfaith Medical Center, New York City, USA

**Keywords:** bradykinin, hoarse voice, methylprednisolone, epinephrine, haloperidol, bipolar disorder, ent, eps, angioneurotic edema, quincke’s disease

## Abstract

Angioneurotic edema is a potentially life-threatening condition characterized by the rapid swelling of subcutaneous and submucosal tissues of the upper respiratory and gastrointestinal tracts. It may result in laryngeal edema, thus obstructing the airway, with a potentially fatal outcome if not diagnosed early. An allergic reaction typically causes it, but certain drugs can induce or contribute to the development of angioneurotic edema in rare cases. Haloperidol is one of the most commonly used antipsychotics to treat psychiatric disorders such as schizophrenia and, in the emergency room, acute delirium and acute psychosis. While it is generally well tolerated, in rare cases, individuals may experience a severe reaction in response to haloperidol administration.

Here, we present the case of angioneurotic edema associated with intramuscular haloperidol use in a 29-year-old female patient with a history of bipolar disorder and no previous history of allergy. This case report aims to raise awareness among clinicians about this rare adverse reaction associated with haloperidol use.

## Introduction

Angioneurotic edema, also known as Quincke's disease, is a potentially life-threatening condition characterized by localized swelling in the subcutaneous and submucosal tissues of the upper respiratory and gastrointestinal tracts [1]. It can be categorized as allergic or non-allergic angioneurotic edema [2]. Non-allergic angioneurotic edema can be further classified into hereditary, acquired, drug-induced, and idiopathic angioneurotic edema. Hereditary angioneurotic edema (HANE) types 1 and 2 are inherited disorders caused by gene mutations affecting the production of C1-inhibitor [2, 3]. In the United States, angioneurotic edema has a lifetime prevalence of around 25%, leading to over one million emergency department visits annually [2, 4]. Allergic reactions account for most cases (approximately 90%) and involve a rapid type 1 hypersensitivity response, activating mast cells and basophils [2]. On the other hand, non-allergic angioneurotic edema, mediated by bradykinin inhibition, often has a delayed onset, occurring hours to days after exposure, and may take several months to manifest [2, 5]. Excessive production of bradykinin, a potent vasodilatory mediator, leads to swelling of the mucosa and submucosal tissues [2, 3, 5].

Symptoms of angioneurotic edema include swelling, particularly around the eyes, lips, and tongue, although it can affect any part of the body, such as the hands, feet, and genitalia [2]. However, patients with Quincke's edema typically present with a sensation of rapidly progressing obstruction or a foreign body in the throat [6]. If the airway is blocked, stridor and noticeable throat swelling are observed [7]. An examination may show a swollen but non-reddened uvula [2, 6]. Additional symptoms may include shortness of breath, dizziness, fainting, and an itchy, raised rash called urticaria (hives), depending on whether the condition is allergic or non-allergic [2, 5, 6]. Drug-induced angioneurotic edema can be triggered by various medications such as angiotensin-converting enzyme inhibitors (ACEIs), angiotensin-2 antagonists, non-steroidal anti-inflammatory drugs (NSAIDs), selective serotonin reuptake inhibitors (SSRIs), oral contraceptive pills (OCPs), proton pump inhibitors (PPIs), vaccines, and rarely by antipsychotics like risperidone, iloperidone, and droperidol [1, 2, 8].

Haloperidol, a first-generation neuroleptic commonly used to treat schizophrenia and manage acute delirium and psychotic agitation in the emergency department, belongs to the butyrophenone class, sharing pharmacological similarities with droperidol derivatives [1]. Its primary mechanism of action is the antagonism of the dopamine-2 receptor, thereby improving positive symptoms of psychosis [9]. Typical side effects of haloperidol include extrapyramidal reactions, neuroleptic malignant syndrome, tremors, dry mouth, weight gain, restlessness, orthostatic hypotension, and tachycardia [1, 9]. Angioneurotic edema is a rare adverse effect of haloperidol, and its exact mechanism of occurrence is not fully understood [1]. It is believed that haloperidol may increase vascular permeability by releasing inflammatory mediators such as bradykinin [1, 10]. During an angioneurotic edema attack, plasma levels of bradykinin significantly increase, reaching up to seven times higher than normal [11]. Unlike histamine-mediated angioedema, antihistamines are ineffective in bradykinin-mediated cases [11, 12].

## Case presentation

A 29-year-old female, a single college student, was brought to the emergency department (ED) by the emergency medical services (EMS) after expressing suicidal ideation. The patient had a history of bipolar disorder and cannabis use disorder. Previous medication trials included aripiprazole (7.5 mg oral daily) for bipolar disorder. The patient reports poor medication compliance with her prescribed medications over the past six months. Before her ED presentation, the patient reported consuming many edibles. However, the urine toxicology test was negative for cannabinoids, barbiturates, benzodiazepines, methadone, amphetamines, opiates, and phencyclidine. Her ethanol level was below 10 mg/dL. The chest X-ray and pregnancy test results were normal. Electrocardiography revealed sinus tachycardia but was otherwise unremarkable. Vital signs indicated normal blood pressure, a heart rate of 118 beats per minute, a normal temperature, and a normal respiratory rate. Oxygen saturation was 98% in room air. Repeat electrocardiography showed a normal sinus rhythm. A complete metabolic panel and other laboratory tests were within the normal range (Table 1).

**Table 1:** The results of the laboratory investigation

Investigations	Results	Normal range
Blood urea nitrogen (BUN)	12.0	6.0-23.0 mg/dL
Creatinine (Cr)	0.60	0.50-1.20 mg/dL
Sodium (Na)	138	136-145 mmol/L
Potassium (K)	4.2	3.5-5.0 mmol/L
Calcium (Ca)	9.2	8.5-10.5 mg/dL
Chloride (Cl)	103	96-106 mmol/L
Magnesium (Mg)	2.2	1.9-2.7 mg/dL
Phosphorous (P)	4.2	2.5-5.0 mg/dL
Osmolality	294	275-295 mOsm/kg
Folate	16.23	5.9-24.8 ng/mL
Vitamin B12	763	180-914 pg/mL
Alanine transaminase (ALT)	20	0-33 U/L
Aspartate transaminase (AST)	25	5-32 U/L
Alkaline phosphatase (ALP)	47	35-104 U/L
White blood count (WBC)	4.5	4.5-11.0 10X3/UL
Lymphocytes	12.9%	22-48%
Monocytes	0.7%	2-14.0%
Eosinophils	0.0%	0.5-5.0%
Neutrophils	86.2%	40-70%
Absolute neutrophil % (ANC)	3.40	2.00-7.9 10X3/UL
Hemoglobin (Hgb)	14.1	12.0-16.0
Hematocrit (HCT)	41.6	39-53%
Platelet (PLT)	406	130-400 10X3/UL
Blood alcohol level (BAL)	<10	0.0-14.0 mg/dL
Urine toxicology (urine tox)	Negative	
Beta hCG quantitative	<0.6	<5 (Negative pregnancy)
Thyroid-stimulating hormone (TSH)	0.656	0.465-4.680 UIU/mL
Free thyroxine (FT4)	1.20	0.61-1.12 ng/dL
Prolactin	45.2	4-15.2 ng/mL
Cholesterol	173	120-200 mg/dL
Triglycerides (TG)	44	<150 mg/dL
High-density lipoprotein (HDL)	74	60-80 mg/dL
Low-density lipoprotein (LDL)	90	50-130 mg/dL
Serum creatinine kinase (CK)	797	30-200 U/L
Hemoglobin A1c (A1c)	5.6	4.8-5.6%
immunoglobulin E (IgE)	24	6-495 IU/mL
C1 esterase inhibitor	30	21-39 mg/dL
Complement C3, serum	113	82-167 mg/dL
Complement C4, serum	26	12-38 mg/dL

Upon assessment in the ED, the patient displayed anxiety symptoms, exhibited bizarre behavior, and showed signs of psychosis, including a disorganized thought process and auditory hallucinations. To manage her psychotic agitation, the patient received intramuscular injections of Haldol (5 mg), Ativan (2 mg), and Benadryl (50 mg). There was no associated history of fever, chills, skin rash, hives, dizziness, upper respiratory symptoms, or trauma. Consequently, the patient was admitted to the psychiatric unit for further management and stabilization. Between 24 and 48 hours following admission, the patient reported symptoms of nausea, neck stiffness, muscle spasms, increased anxiety, a choking sensation in her throat, and difficulty speaking. Physical examination revealed mild facial swelling, mild uvula edema, and a hoarse voice. The patient did not report signs of urticaria or pruritus, and the patient's vital signs remained stable, saturating 100% on room air and without signs of respiratory distress. The patient denied any known medication allergies and claimed to have never experienced a similar episode after receiving intramuscular or oral haloperidol. The patient also denied taking any medications, including angiotensin-converting enzyme inhibitors (ACEIs), angiotensin-2 receptor antagonists, non-steroidal anti-inflammatory drugs (NSAIDs), selective serotonin reuptake inhibitors (SSRIs), oral contraceptive pills (OCPs), proton pump inhibitors (PPIs), antipsychotics, or being vaccinated recently before her admission.

The patient was promptly administered benztropine (2 mg) intramuscularly as a STAT dose to address possible extrapyramidal symptoms (EPS). Additionally, oral benztropine (2 mg) was prescribed to prevent further EPS. Shortly after receiving the intramuscular benztropine, the patient experienced a near-fall episode, prompting a transfer to the medical floor. There was no evidence of seizure-like episodes.

**Figure 1:**
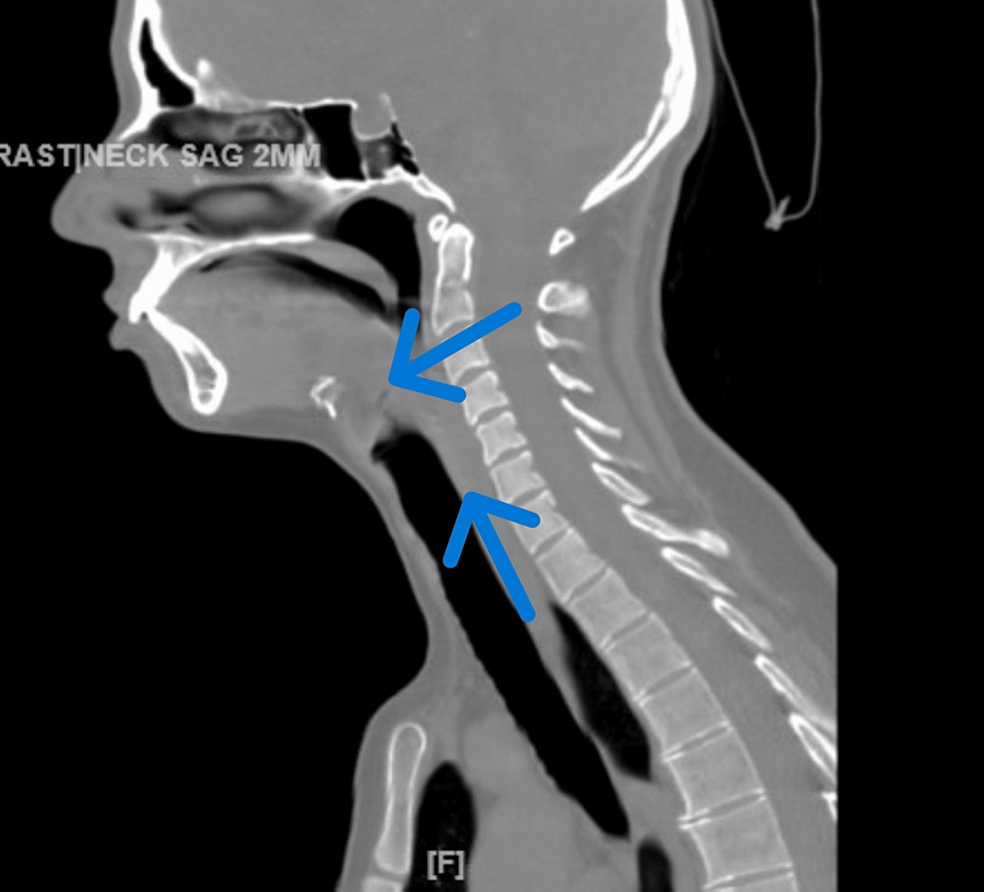
A CT scan of the soft tissues of the neck (without contrast) during transfer to the medical floor showed extensive soft tissue swelling within the larynx and hypopharynx

Prior electroencephalograms (EEGs) retrieved from her chart revealed no epileptiform activity. Upon transfer, the patient received a STAT dose of oral Depakote (1 g), followed by 250 mg twice daily per neurology recommendation, to address the possibility of withdrawal seizures from her substance use. Neurological examination yielded negative findings for stroke, and a head CT scan showed no signs of acute transcortical infarct, acute intracranial hemorrhage, or mass effect. However, a CT scan of the soft tissues in the neck revealed extensive swelling within the hypopharynx and larynx, resulting in airway obstruction at the level of the hyoid cartilage, as shown in Figure 1.

Her white blood cell count (WBC) was normal; however, abnormalities in her differentials could be acute stress-induced. Other laboratory investigations, including complete blood counts, renal and hepatic function tests, and serum complement levels, were all within normal limits, as shown in Table 1, except for an elevated serum creatinine kinase level, possibly due to dehydration or recreational drug use.

Considering the possibility of an allergic etiology, the ENT team recommended emergency administration of methylprednisolone 125 mg, followed by 60 mg every eight hours intravenously for the next 24 hours, and epinephrine 0.30 mg intramuscularly once, followed by as-needed doses. The patient was treated with intramuscular epinephrine and intravenous corticosteroids. Within minutes, the patient exhibited improvement in facial swelling, uvula edema, nausea, neck stiffness, anxiety, choking sensation, and voice quality.

**Figure 2:**
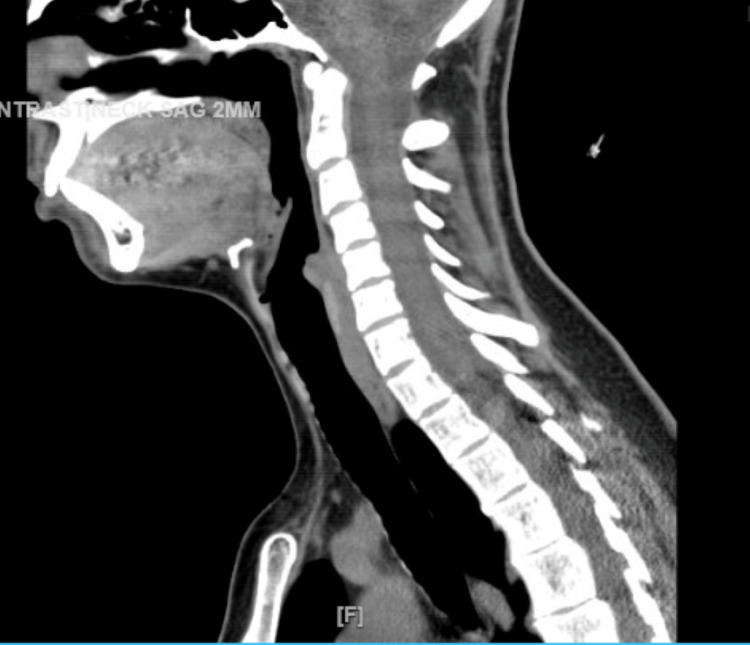
A CT scan of the soft tissues of the neck (without contrast) during discharge from the medical floor

Due to the possibility of drug-induced angioedema, haloperidol was discontinued, and the patient was closely monitored over the next 24-72 hours. There was complete resolution of her edema of the face and uvula. A repeat CT scan of the soft tissues of the neck without contrast revealed a marked resolution of edema within the larynx and hypopharynx, as shown in Figure 2.

The patient reported significant improvement in her voice quality, leading to her subsequent transfer to the inpatient psychiatry floor with recommendations for alternative antipsychotic medications after discussing the risks and benefits.

## Discussion

Drug-induced angioneurotic edema is a rare, severe reaction that can occur with certain medications, including antipsychotics [1, 5, 8, 10]. It is a life-threatening condition characterized by rapid and deep swelling in the skin and tissues, resulting in non-pitting edema [1, 4, 6]. While it commonly affects the lips, face, tongue, and throat, it can also involve other body parts, such as the limbs, genitals, and gastrointestinal tract [2, 3, 10]. This sudden swelling can lead to airway obstruction within minutes and may take hours or days to resolve [4, 10, 11].

Haloperidol, a first-generation antipsychotic, has been associated with rare cases of angioneurotic edema [1]. The exact frequency of haloperidol-induced angioneurotic edema is not well established, but healthcare providers should be aware of this potential reaction, particularly in patients taking haloperidol [1,10]. 

The Naranjo scale, a validated tool used to evaluate the likelihood of a drug causing an adverse reaction, utilizes various criteria to determine the probability of a causal relationship. It is reported based on definite, probable, possible, and doubtful criteria [13]. Positive items on the Nanjaro scale assessment for the patient include: Are there previous conclusive reports on this reaction? (1+); Did the adverse event appear after the suspected drug was administered? (2+); Did the adverse event improve when the drug was discontinued or a specific antagonist was administered? (1+); Did any other objective evidence confirm the adverse event? (1+).

In this case, the medication-angioneurotic edema relationship was classified as "probable" on the Naranjo scale. However, there were no changes in medication or the introduction of other substances that could explain the patient's condition. It is also important to note that there was no previous history of angioneurotic edema symptoms before or after stopping the haloperidol. Other factors that could potentially complicate the situation include the consumption of edibles. Urine toxicology was negative, and screening for synthetic cannabinoids was not performed, which could rule out synthetic cannabis ingestion.

Additionally, only one case report has cited isolated uvula edema in a 28-year-old female without pharyngeal or laryngeal involvement, which was reported after smoking marijuana and caused irritation of the mucous membranes, as marijuana smoking burns at a higher temperature than tobacco [14]. Other cases have reported improved symptoms with cannabis treatment in refractory cases of idiopathic angioneurotic edema [15, 16]. The mechanism by which it works is not fully understood. However, it is believed that cannabinoids produce anti-inflammatory effects by inhibiting pro-inflammatory cytokines and upregulating anti-inflammatory cytokines [16].

In our case, a 29-year-old female diagnosed with bipolar disorder experienced sudden swelling of the face, hoarseness, and difficulty speaking. The patient reported symptoms of nausea, neck stiffness, muscle spasms, increased anxiety, a choking sensation in her throat, and difficulty speaking. However, the patient was promptly administered 2 mg of benztropine intramuscularly as a STAT dose to address possible extrapyramidal symptoms (EPS). Due to the subjectivity of the symptoms reported by the patients, the initial treatment is usually targeted toward a dystonic reaction [10]. The patient was later transferred to the medical floor following a near-fall episode after receiving intramuscular benztropine, thus prompting further investigations.

The absence of hives and itching ruled out an allergic cause, and the 24-36 hour timing between receiving a haloperidol injection and the onset of symptoms suggested non-allergic angioneurotic edema [5, 12]. Although most patients develop reactions shortly after starting the medications, the reaction may persist for up to three months after the drug withdrawal [2, 4, 6, 11, 12]. Laboratory tests, including blood count, kidney and liver function tests, IgE test, and complement levels, were within normal ranges, as shown in Table 1, eliminating other potential causes of non-allergic angioneurotic edema. Allergy evaluation, family history assessment, and complement tests like C4 level, C-1 esterase inhibitor, IgE, and serum mast cell tryptase can help rule out hereditary and allergic causes of angioneurotic edema [3, 5, 11]. In the case of anaphylaxis, the serum mast cell tryptase (MCT) level is normal in hereditary angioedema but elevated in anaphylaxis and other mast cell disorders associated with angioedema [3, 4, 17]. Quincke's edema is quite different from uvulitis, an infectious condition frequently induced by or occurring along with inflammation of the nearby epiglottis [3, 6].

For this reason, direct visualization of the throat or imaging by neck X-rays or other imaging, such as a computerized tomography (CT) scan, should be done as needed to rule out uvulitis [7]. In our case report, a CT scan of the neck revealed significant swelling in the hypopharynx and larynx, leading to airway obstruction at the level of the hyoid cartilage, as shown in Figure 1. Flexible fiberoptic laryngoscopy may be necessary to assess the involvement of the tongue and larynx in patients with symptoms affecting the head, neck, and upper airway, requiring a prompt referral to an ear, nose, and throat (ENT) specialist [18].

Managing haloperidol-induced angioneurotic edema involves promptly recognizing the condition, discontinuing the medication, administering epinephrine, corticosteroids, and antihistamines, and providing supportive care [1, 4, 5, 10]. The severity of the reaction can also vary, with some cases spontaneously resolving, whereas others rapidly progress to airway compromise [4, 6, 8, 10]. In our case, we quickly stopped the drug upon suspecting haloperidol as the cause and closely monitored the patient. The symptoms gradually improved within a few minutes after administering corticosteroids and epinephrine. We observed our patient for 24-72 hours before she was transferred back to the inpatient psychiatric floor with recommendations for alternative antipsychotic medications.

The exact mechanism of haloperidol-induced angioneurotic edema still needs to be fully understood. However, haloperidol, like other antipsychotics, may increase the permeability of blood vessels, potentially by releasing inflammatory substances like bradykinin, a potent transient vasodilator resulting in the extravasation of fluid into the extracellular space and thereby causing soft-tissue edema [1, 10]. Further research is needed to better understand the underlying processes involved in haloperidol-induced angioneurotic edema and identify potential risk factors for its development.

## Conclusions

Clinicians should remain vigilant when recognizing signs and symptoms of angioneurotic edema in patients taking antipsychotic medications such as haloperidol. Prompt diagnosis and management are crucial in preventing airway compromise and other serious complications. Discontinuation of the causative agent, administration of epinephrine, corticosteroids, and antihistamines, and close monitoring are essential steps in managing haloperidol-induced angioneurotic edema. However, given the potential risk of airway compromise, some patients may require intubation. Prescribing safe alternative antipsychotic medications to these patients after discussing the benefits and risks with them is also crucial. Collaborating with specialists, such as ENT specialists and allergists, can help ensure appropriate evaluation and management in such cases.
